# Flash Joule Heating: A Promising Method for Preparing Heterostructure Catalysts to Inhibit Polysulfide Shuttling in Li–S Batteries

**DOI:** 10.1002/advs.202405351

**Published:** 2024-07-16

**Authors:** Huiyi Dong, Lu Wang, Yi Cheng, Huiyue Sun, Tianqi You, Jingjing Qie, Yifan Li, Wuxing Hua, Ke Chen

**Affiliations:** ^1^ Center for the Physics of Low‐Dimensional Materials Henan Joint International Research Laboratory of New Energy Materials and Devices School of Physics and Electronics Henan University Kaifeng 475004 China; ^2^ School of Materials Science and Engineering Shandong University Jinan 250061 China; ^3^ Center for Nanochemistry, Beijing Science and Engineering Center for Nanocarbons, Beijing National Laboratory for Molecular Sciences, College of Chemistry and Molecular Engineering Peking University Beijing 100871 China

**Keywords:** electrocatalysis, internal electric field, lithium–sulfur battery, shuttle effect, W‐W_2_C heterostructure

## Abstract

The “shuttle effect” issue severely hinders the practical application of lithium–sulfur (Li–S) batteries, which is primarily caused by the significant accumulation of lithium polysulfides in the electrolyte. Designing effective catalysts is highly desired for enhancing polysulfide conversion to address the above issue. Here, the one‐step flash‐Joule‐heating route is employed to synthesize a W‐W_2_C heterostructure on the graphene substrate (W‐W_2_C/G) as a catalytic interlayer for this purpose. Theoretical calculations reveal that the work function difference between W (5.08 eV) and W_2_C (6.31 eV) induces an internal electric field at the heterostructure interface, accelerating the movement of electrons and ions, thus promoting the sulfur reduction reaction (SRR) process. The high catalytic activity is also confirmed by the reduced activation energy and suppressed polysulfide shuttling by in situ Raman analyses. With the W‐W_2_C/G interlayer, the Li–S batteries exhibit an outstanding rate performance (665 mAh g^−1^ at 5.0 C) and cycle steadily with a low decay rate of 0.06% over 1000 cycles at a high rate of 3.0 C. Moreover, a high areal capacity of 10.9 mAh cm^−2^ (1381.4 mAh g^−1^) is obtained with a high area sulfur loading of 7.9 mg cm^−2^ but a low electrolyte/sulfur ratio of 9.0 µL mg^−1^.

## Graphical abstract

A W‐W_2_C heterostructure catalyst is effectively synthesized via the flash‐Joule‐heating technique. The work function difference between W and W_2_C induces the spontaneous generation of an internal electric field at their heterostructure interface, which subsequently facilitates the movement of electrons and ions, thereby enhancing the sulfur reduction reaction process to inhibit the shuttle effect.

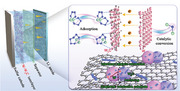

## 1 Introduction

Due to the high energy density (2600 Wh Kg^−1^), low cost, and environmental friendliness, lithium–sulfur (Li–S) batteries have garnered widespread attention as one of the promising next‐generation energy storage systems.^[^ ^1^, ^2^, ^3^ ^]^ However, the sluggish conversion from intermediate lithium polysulfides (LiPSs, also denoted Li_2_S*_n_*, 4 ≤ *n* ≤ 8) to the discharge products (Li_2_S_2_/Li_2_S) leads to LiPS accumulation in organic electrolytes, where the soluble LiPSs tend to migrate between electrodes under the concentration gradients, leading to the so‐called “shuttle effect” issue. This results in low utilization of sulfur and fast capacity decay, hindering the practical applications of Li–S batteries.^[^ ^4^, ^5^, ^6^, ^7^ ^]^

To address the aforementioned challenges, the strategies of physical adsorption and chemical anchoring have been used to confine or block LiPSs among the cathode side,^[^ ^8^, ^9^, ^10^ ^]^ which are both intrinsically passive solutions as they do not effectively suppress the dissolution and accumulation of LiPSs.^[^ ^11^ ^]^ In the solid–liquid–solid sulfur reduction reaction (SRR) process, the kinetics difference of each step results in the inability of generated LiPSs to rapidly convert into insoluble Li_2_S_2_/Li_2_S, which is the fundamental cause of the shuttle effect.^[^ ^11^, ^12^, ^13^, ^14^ ^]^ Recently, a p charge descriptor was obtained to predict the SRR activity and the maximum p charge gain achieved by Bi_2_S_3_ exhibited the lowest energy barrier of the rate‐determining step (RDS, the conversion of LiPSs to Li_2_S_2_/Li_2_S) among the p‐block metal sulfides, which greatly improved the battery performance.^[^ ^15^ ^]^ Therefore, exploring highly active catalysts to decrease the energy barrier of RDS is crucial for inhibiting the shuttle effect. The catalytic conversion of SRR involves an adsorption–conversion–deposition process, which remains challenging for single‐component catalysts. For example, metal oxides, like TiO_2_, VO_2_, etc., exhibit strong adsorption of LiPSs but limited catalytic conversion activity, while metal nitrides including TiN, VN, etc., possess higher catalytic activity but weaker absorption capabilities toward LiPSs.^[^ ^16^, ^17^ ^]^ Therefore, various heterostructures with two or more components, such as WS_2_‐WO_3_, TiO_2_‐TiN, MoN‐VN, Co‐MoN, and Bi‐Bi_2_O_3_ have been screened to balance the adsorption–conversion–deposition process of SRR, thus inhibiting the shuttle effect.^[^ ^18^, ^19^, ^20^, ^21^, ^22^, ^23^ ^]^ According to previous reports, tungsten catalyst exhibits excellent catalytic activity, promoting the nucleation and decomposition of Li_2_S and thereby endowing Li–S batteries with an outstanding rate performance.^[^ ^24^ ^]^ Additionally, the polar ditungsten carbide (W_2_C) shows strong adsorption capabilities for the soluble LiPSs, avoiding their diffusion into the electrolyte to suppress the shuttle effect.^[^ ^25^ ^]^

**Figure 1:**
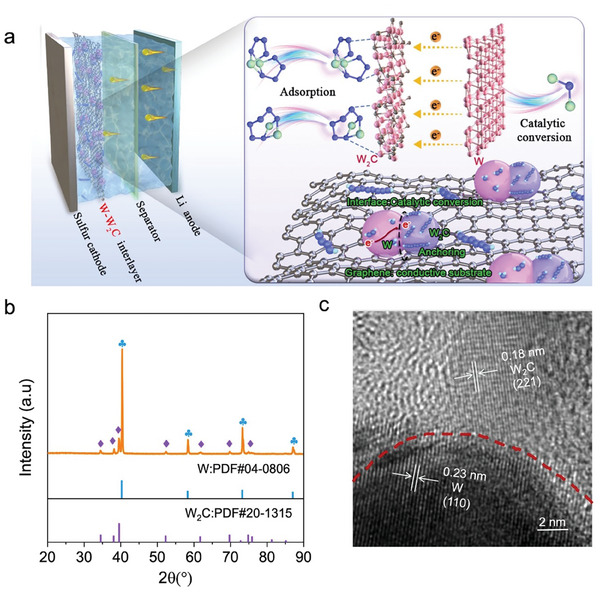
Catalytic mechanism and materials characterization. a) The mechanism diagram of adsorption and catalytic conversion of LiPSs on W‐W_2_C heterostructure interface. b) XRD patterns of W‐W_2_C/G heterostructure. c) HRTEM images of the as‐prepared W‐W_2_C/G heterostructure.

Leveraging the advantages of W and W_2_C, here, a rapid synthesis of W‐W_2_C heterostructure (denoted, W‐W_2_C/G) catalyst is demonstrated using the flash‐Joule‐heating method in macroscopic quantity. The W‐W_2_C/G catalyst serves as a catalytic interlayer coating on the carbon nanotubes/sulfur cathode (denoted, CNTs/S@W‐W_2_C/G) to intercept LiPSs and accelerate the RDS process, promoting the nucleation and growth of Li_2_S. The CNTs are used as the sulfur host due to their high conductivity and high specific surface area.^[^ ^10^ ^]^ Density functional theory (DFT) calculations indicate that the smaller work function of W (5.08 eV) than that of W_2_C (6.31 eV) drives electrons to flow from W to W_2_C thus creating a spontaneous internal electric field at the interface. In this process, as illustrated in **Figure** **1**a, the graphene serves as the conductive substrate, W_2_C functions as the anchor for LiPSs, and the generated internal electric field can accelerate electron transfer and ion diffusion. This configuration optimizes the interface electron states of the W‐W_2_C heterostructure to enhance the adsorption and conversion of LiPSs, thus effectively suppressing the LiPS shuttling. The accelerated SRR process is also confirmed by the reduced activation energy, promising low overpotentials and high Li_2_S deposition capacity, as well as excellent cycle performance. As a result, a slow capacity decay rate of 0.06% per cycle is realized at a high rate of 3.0 C within 1000 cycles, and a high‐capacity retention of 80.6% after 100 cycles at 0.2 C is achieved even with a high sulfur loading of 7.9 mg cm^−2^ in the participation of W‐W_2_C/G heterostructure.

## 2 Results and Discussion

### 2.1 Fabrication and Characterization of W‐W_2_C/G Heterostructure Catalyst

The W‐W_2_C/G heterostructure catalysts are prepared through the flash Joule heating method with the advantage of in situ rapid synthesis in macroscopic quantity.^[^ ^26^ ^]^ The synthesis mechanism is illustrated in Figure S1 (Supporting Information), where the ultrafast heating/cooling, along with the short growth time, ensured the uniform dispersion of W‐W_2_C/G nanoparticles with numerous W‐W_2_C/G heterostructure interfaces, which can effectively avoid the catalytic activity decay caused by aggregation.^[^ ^27^ ^]^ The peaks in the X‐ray diffraction pattern could be indexed as cubic tungsten (W) (PDF#04‐0806) and orthorhombic tungsten carbide (W_2_C) (PDF# No. 20‐1315), respectively (Figure 1b).

Figures S2 and S3 (Supporting Information) display scanning electron microscope (SEM) images of the synthesized W‐W_2_C/G catalyst, along with corresponding high‐angle annular dark‐field (HAADF) images and elemental maps. The results reveal a uniform distribution of W and C on the graphene substrate. Combined with transmission electron microscope (TEM) analysis (Figure S4, Supporting Information), it can be inferred that the macroscopically distributed W‐W_2_C particles have anchored on the graphene surface. Additionally, the TEM image reveals a thin W_2_C coating layer on W with the clear interface, suggesting the surface carbonization of the W to form the W‐W_2_C heterostructure. The high‐resolution TEM image (Figure 1c) demonstrates two lattice fringes with spacings of 0.23 and 0.18 nm, corresponding to the (110) plane of W and the (221) plane of W_2_C, respectively. Besides, an explicit interface between W and W_2_C can be observed, suggesting the successful construction of W‐W_2_C heterostructure. Such an interface spontaneously generates an internal electric field, enabling fast charge transfer for accelerating LiPS conversions.

### 2.2 Kinetics of the SRR over W‐W_2_C/G Heterostructure Catalyst

**Figure 2:**
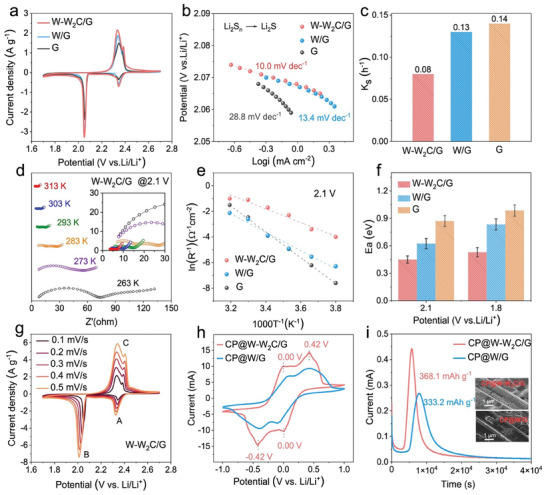
Electrocatalysis. a) CV curves of Li–S batteries with W‐W_2_C/G, W/G, and G interlayers (sulfur loading: 1.0 mg cm^−2^) at a scan rate of 0.1 mV s^−1^. b) Tafel plots corresponding to the reduction of Li_2_S*_n_* to Li_2_S. c) Shuttle constants in the batteries with W‐W_2_C/G, W/G, and G interlayers. d) EIS measurements at various temperatures at 2.1 V for the battery with W‐W_2_C/G interlayer; The error bars represent the standard deviation. e) Arrhenius plots for the three batteries calculated from the EIS curves at 2.1 V. f) Activation energies for the three batteries at 2.1 and 1.8 V, as calculated from e. g) CV curves of the battery with W‐W_2_C/G interlayer at scan rates of 0.1, 0.2, 0.3, 0.4, 0.5 mV s^−1^. h) CV curves of Li_2_S_6_–Li_2_S_6_ symmetric batteries at 5 mV s^−1^. i) Potentiostatic discharge curves of the Li_2_S deposition tests using W‐W_2_C/G and W/G as substrates (the insets are SEM images of the Li_2_S deposition morphology on different substrates).

To evaluate the catalytic activity of the as‐synthesized W‐W_2_C/G heterostructure, cyclic voltammetry (CV) curves for the batteries with W‐W_2_C/G, W/G, and G interlayers were compared in **Figure** **2**a, which were respectively assembled by coupling CNTs/S@W‐W_2_C/G, CNTs/S@W/G and CNTs/S@G cathodes with lithium metal as anodes. In the SRR process, two reduction peaks at 2.34 and 2.05 V are attributed to the conversion of S_8_ to soluble LiPSs and subsequent reduction to insoluble Li_2_S_2_/Li_2_S, respectively. For the charging process, two oxidation peaks around 2.3–2.4 V represent the reversible oxidation of Li_2_S/Li_2_S_2_ to LiPSs and the eventual formation of S_8_.^[^ ^5^, ^15^ ^]^ Notably, the battery with the W‐W_2_C/G interlayer shows larger current density in both reduction and oxidation peaks than those of the other two batteries, confirming the enhanced redox reactions with the W‐W_2_C/G heterostructure catalyst.^[^ ^11^, ^28^ ^]^

To further assess the conversion kinetics of the SRR, the Tafel slope was calculated based on the CV curves.^[^ ^29^, ^30^ ^]^ As shown in Figure S5 (Supporting Information), the battery with the W‐W_2_C/G interlayer exhibits the smallest Tafel slope among the three batteries, indicating that the W‐W_2_C/G catalyst promotes the reduction of S_8_. During the reduction of LiPSs to Li_2_S_2_/Li_2_S (RDS), the Tafel slope value for the battery with W‐W_2_C/G interlayer (10.0 mV dec^−1^) is smaller than that for the batteries with W/G (13.4 mV dec^−1^) and G (28.8 mV dec^−1^) interlayers (Figure 2b), demonstrating the efficient catalytic activity of the W‐W_2_C/G heterostructure and the optimized conversion kinetics of the RDS in SRR process.^[^ ^31^, ^32^, ^33^ ^]^ The charge efficiency that is used to estimate the shuttle constant (*k*_s_) obtained from the rate performance (Figures S6 and S7, Supporting Information), is much lower for the battery with W‐W_2_C/G interlayer (0.08 h^−1^) compared to that for the batteries with W/G interlayer (0.13 h^−1^) and G interlayer (0.14 h^−1^) (Figure 2c), which indicates that the W‐W_2_C/G heterostructure effectively suppresses the shuttle effect, enhancing the utilization of sulfur.^[^ ^12^, ^15^ ^]^

The SRR kinetics at a given voltage represented by *E*_a_, were determined experimentally by fitting the charge transfer resistance, probed at various temperatures via electrochemical impedance spectroscopy (EIS), to the Arrhenius equation (Figure 2d–f and Figures S8 and S9, Supporting Information).^[^ ^14^, ^15^ ^]^ In order to maintain a consistent voltage during a particular conversion process, the cell was discharged to the specified potential and maintained at that level (chronoamperometry) until the EIS measurements were taken at different temperatures. The temperature was controlled through a variable temperature thermotank. The tail of the Nyquist plots for the three batteries at 2.1 V is the Warburg resistance, and the semicircle represented by *R*_ct_ belongs to the charge transfer process (SRR process) at the catalyst surface.^[^ ^14^ ^]^ The calculated *E*_a_ at 2.1 V was 0.4 eV for the battery with W‐W_2_C/G interlayer, which is much lower than that for the batteries with W/G interlayer (0.6 eV) and G interlayer (0.9 eV), indicating the accelerated conversion from soluble LiPSs to short‐chain LiPSs with W‐W_2_C/G catalyst. When the voltage decreases to 1.8 V, corresponding to the conversion of LiPSs to Li_2_S_2_/Li_2_S of SRR, the *E*_a_ is slightly increased to 0.5 eV for the battery with the W‐W_2_C/G interlayer. However, the *E*_a_ at 1.8 V for the batteries with the W/G and G interlayers is markedly increased to 0.8 eV and 1.0 eV, respectively. In this case, the high *E*_a_ for the formation of Li_2_S_2_/Li_2_S would result in the accumulation of LiPSs in the electrolyte and thus the shuttle effect, reducing the utilization of sulfur. The significant decrease of *E*_a_ for the conversion of soluble LiPSs to insoluble Li_2_S_2_/Li_2_S at 1.8 V greatly reduces the accumulation of LiPSs and their diffusion to the Li anode in the presence of the W‐W_2_C/G catalyst. The above results indicate that the energy barrier for LiPS conversion is higher in the battery with only a G interlayer than that for the batteries with W‐W_2_C/G and W/G interlayers. Therefore, the batteries with W/G and W‐W_2_C/G interlayers are emphatically investigated in the following electrochemical analysis.

As lithium‐ion (Li^+^) diffusion plays a crucial role in LiPS conversion, CV measurements (Figure 2g and Figure S10, Supporting Information) were performed for the batteries with W‐W_2_C/G and W/G interlayers at different scan rates (0.1–0.5 mV s^−1^) to assess the Li^+^ diffusion coefficient (*D*_Li+_).^[^ ^34^, ^35^ ^]^ The peak current data is analyzed based on the Randles‐Sevcik equation (1), where *I*_p_ is the peak current (A), *n* is the number of electrons during the reaction, *A* refers to the area of the sulfur cathode, *C*_Li+_ is the Li^+^ concentration, and *V* represents the scan rate.^[^ ^36^ ^]^

(1) $$I_{p}=2.69\times 10^{5}n^{1.5}AD_{Li^{+}}^{0.5}C_{Li^{+}}V^{0.5}$$

Linear fitting of the peak current of the batteries with W‐W_2_C/G and W/G interlayers is shown in Figure S11 (Supporting Information). The higher *D*_Li+_ is achieved for the battery with W‐W_2_C/G interlayer in both reduction peaks and oxidation peaks, which is further evidenced by the galvanostatic intermittent titration technique (GITT) results (Figure S12, Supporting Information).^[^ ^37^ ^]^ These results demonstrate a significant improvement in the diffusion rate of Li^+^ and charge transfer efficiency over the W‐W_2_C/G catalyst, which is essential for enhancing the electrochemical performance of Li–S batteries, especially under high sulfur loading and low electrolyte conditions.^[^ ^38^ ^]^

To investigate the catalytic capability of the W‐W_2_C/G heterostructure in promoting polysulfide conversion, symmetrical cells were assembled using two same electrodes (W‐W_2_C/G loaded on carbon fiber paper, denoted CP@W‐W_2_C/G) and an electrolyte containing Li_2_S_6_. As shown in Figure 2h, the CP@W‐W_2_C/G cell exhibits two evident reduction peaks at 0.00 V and −0.42 V, representing the reduction of S_8_ to LiPSs and then to Li_2_S, and two oxidation peaks at 0.00 and 0.42 V, referring to the reversible conversion of Li_2_S to LiPSs and the further oxidation to S_8_. However, the two pairs of redox peaks in the CV curves of the CP@W/G cell are not obvious and much weaker than those of the CP@W‐W_2_C/G cell. This result confirms a significant improvement in the redox kinetics of LiPSs in the presence of W‐W_2_C/G catalysts.^[^ ^39^, ^40^ ^]^ The conversion kinetics of LiPSs to Li_2_S was evaluated through potentiostatically Li_2_S deposition experiments.^[^ ^41^, ^42^ ^]^ The cells for the tests were assembled using CP@W‐W_2_C/G or CP@W/G as the cathodes, 20 µL Li_2_S_8_ solution as the catholyte, and Li foil as the anode. As shown in Figure 2i and Figure S13 (Supporting Information), the peak current of the CP@W‐W_2_C/G cell appears much earlier (≈5700 s) than that of the CP@W/G cell (≈7800 s). Besides, the CP@W‐W_2_C/G cell shows a higher Li_2_S precipitation capacity of 368.1 mAh g^−1^ than that for the CP@W/G cell (333.2 mAh g^−1^), thus more Li_2_S precipitates on the CP@W‐W_2_C/G substrate (the inset of Figure 2i). Li_2_S precipitation test indicates that the W‐W_2_C/G catalyst significantly improves the conversion kinetics of LiPSs to Li_2_S, consistent with the above *E*_a_ calculations obtained from the temperature‐dependent EIS measurements. Furthermore, the dissolution of deposited Li_2_S was investigated by using a potentiostatic charging process (Figure S14, Supporting Information). The dissolution capacity estimated by the quantity of electric charge is much higher for the CP@W‐W_2_C/G cell (1122.5 mAh g^−1^) compared to that of the CP@W/G cell (868.2 mAh g^−1^), indicating the effective oxidation of Li_2_S on the W‐W_2_C/G surface. Moreover, the oxidation current density exhibits a notable increase in the CP@W‐W_2_C/G cell, indicating enhanced efficiency in Li_2_S dissolution. The higher capacities for Li_2_S precipitation/dissolution with the CP@W‐W_2_C/G catalyst compared to the CP@W/G catalyst further support the superior bidirectional catalytic activity of the W‐W_2_C/G catalyst in redox reactions.^[^ ^43^ ^]^

### 2.3 Theoretical Calculations

**Figure 3:**
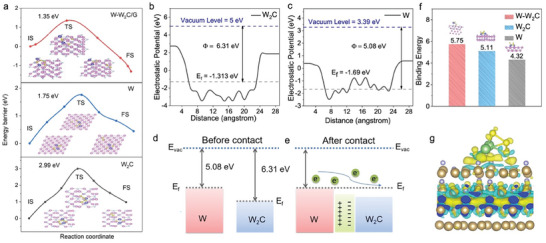
Theoretical calculations. a) The energy profiles involving initial‐transition‐final states for conversion of Li_2_S_4_ on W_2_C, G, and W‐W_2_C. The calculated electronic potential of b) W_2_C and c) W. Schematic diagrams of band structure for W and W_2_C d) before and e) after their contact. f) The adsorption energy of Li_2_S_4_ on W_2_C, G and W‐W_2_C. g) The electron density difference of Li_2_S_4_ on W‐W_2_C.

Theoretical calculations based on DFT are conducted to further probe the intrinsic features of W‐W_2_C to enhance its catalytic performance for reactions of sulfur‐related species conversion. As shown in **Figure** **3**a, the LiPSs on the W‐W_2_C heterostructure exhibit a conversion energy of 1.35 eV, which is observably lower than that of the other two catalysts (1.75 eV of W and 2.99 eV of W_2_C). The work function of W and W_2_C has been also calculated to be 5.08 and 6.31 eV, respectively (Figure 3b,c), and the band structure of W and W_2_C shown in Figure 3d,e vividly demonstrate the construction of built‐in electric field between W and W_2_C, which could facilitate the charge transfer for the electrochemical reactions in sulfur cathode withW‐W_2_C heterostructure catalyst. The adsorption energy shown in Figure 3f indicates that the construction of heterostructure has significantly enhanced the interaction between Li_2_S_4_ and W‐W_2_C heterostructure in comparison with the individual W or W_2_C cases, and the origin of its high adsorption energy is unveiled by the charge density difference (Figure 3g), where the yellow region delegates the electron accumulation and the green indicates the electron depletion. Above all, the high catalytic activity of the W‐W_2_C catalyst is attributed to their high adsorption energy towards LiPSs, together with the internal electric field at the heterostructure interface.

### 2.4 Battery Performance

**Figure 4:**
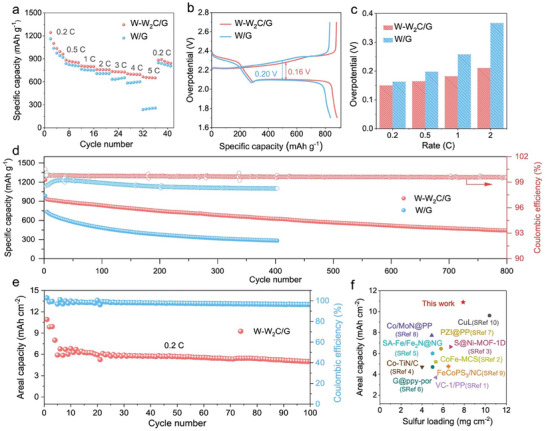
Electrochemical performance. a) Rate performance. b) Galvanostatic discharge/charge profiles at 0.5 C. c) Overpotentials calculated from the voltage difference between the charge and discharge plateaus in a cycle at rates of 0.2,0.5,1.0, and 2.0 C. d) Cycling performance of the batteries with W‐W_2_C/G and W/G interlayers at 1.0 C (sulfur loading: 1.0 mg cm^−2^). e) Cycling performance for battery with W‐W_2_C/G interlayer with a high sulfur mass loading of 7.9 mg cm^−2^ at 0.2 C. f) A comparison with other sulfur cathodes (SRef: Supporting Information References).

The rate performance of Li–S batteries assembled with CNTs/S@W‐W_2_C/G or CNTs/S@W/G as the cathode and lithium metal as the anode was conducted to evaluate the sulfur redox chemistry. With the enhancement of C‐rates, the difference in capacity for the two batteries becomes increasingly noteworthy (**Figure** **4**a). The battery with the W‐W_2_C/G interlayer maintained stable capacities of 736, 706, and 665 mAh g^−1^ at rates of 3.0, 4.0, and 5.0 C, much higher than that for the battery with the W/G interlayer. The corresponding charge–discharge curves of the batteries at various rates (0.2–5.0 C) are illustrated in Figure S15 (Supporting Information). Notably, the battery with W‐W_2_C/G interlayer still performed two extinct discharge plateaus at a high rate of 5.0 C, while the second plateau for the battery with W/G interlayer, corresponding to the reduction of Li_2_S_n_ to Li_2_S_2_/Li_2_S, cannot be observed, confirming the accelerated conversion of RDS with the W‐W_2_C/G catalyst.^[^ ^11^ ^]^ In addition, the overpotentials of the battery with the W‐W_2_C/G interlayer, which are calculated based on the voltage difference between the charge and discharge plateaus, are lower than those of the control sample at all rates (Figure 4b,c). Furthermore, the differential plots corresponding to the charge/discharge profiles have been analyzed for the batteries with W‐W_2_C/G and W/G interlayers (Figure S16, Supporting Information), further revealing a lower overpotential of the battery with a W‐W_2_C/G interlayer compared to the W/G interlayer. The lower overpotentials suggest that the electrochemical kinetics are more favorable over the W‐W_2_C/G catalyst.^[^ ^15^, ^44^ ^]^

The cycling performances of the two types of batteries are further compared at 1.0 and 3.0 C. As shown in Figure 4d, the battery with the W‐W_2_C/G interlayer exhibits a high initial capacity of 929.5 mAh g^−1^ at 1.0 C, and a low capacity decay rate of 0.06% per cycle is obtained after 800 cycles; the initial capacity and its capacity decay rate are much superior to those of the control sample. Besides, the Coulombic efficiency of the battery with W‐W_2_C/G interlayer after 800 cycles is kept at 99.6%, much higher than that of the control sample (98.2%) for 400 cycles, indicating that the shuttle effect is greatly suppressed due to the enhanced conversion of LiPSs with the W‐W_2_C/G catalyst.^[^ ^45^ ^]^ As the current density is increased to 3.0 C, the battery with the W‐W_2_C/G interlayer still demonstrates an outstanding cycling performance with a capacity decay rate as low as 0.06% per cycle over 1000 cycles. In sharp contrast, the capacity quickly decays to 324 mAh g^−1^ with a decay rate of 0.13% after 400 cycles for the control sample (Figure S17, Supporting Information). These results indicate that the W‐W_2_C/G catalyst effectively enhances sulfur utilization, greatly improving the longevity of Li–S batteries.

To further demonstrate the high activity of the W‐W_2_C/G for catalytic conversion of LiPSs, high‐loading sulfur cathodes were tested under a low electrolyte/sulfur ratio of 9.0 µL mg^−1^. As shown in Figure S18 (Supporting Information), when the sulfur mass loading is increased to 7.6 mg cm^−2^, a high initial areal capacity of 10.6 mAh cm^−2^ is obtained for the battery with the W‐W_2_C/G interlayer at a current of 0.02 C, much higher than that for the battery with W/G interlayer (8.0 mAh cm^−2^). When the current density is increased to 0.1 C, the battery with the W‐W_2_C/G interlayer can still deliver 7.3 mAh cm^−2^, while the battery with the W/G interlayer almost cannot normally operate at this current. The cycling performance of the battery with the W‐W_2_C/G interlayer was carried out at 0.2 C. As shown in Figure 4e, a high initial areal capacity of 10.9 mAh cm^−2^ is achieved at 0.02 C, and an areal capacity of 5.0 mAh cm^−2^ is maintained over 100 cycles, representing a high capacity retention of 80.6%. The corresponding charge and discharge plateaus are clearly seen even at such a high sulfur loading (Figure S19, Supporting Information). Overall, the areal capacities of the battery with W‐W_2_C/G interlayer at high sulfur loadings are remarkable in comparison with those for previous reported Li–S batteries (Figure 4f and Table S1, Supporting Information).

### 2.5 In Situ Raman Measurement for Monitoring the Shuttle Effect

**Figure 5:**
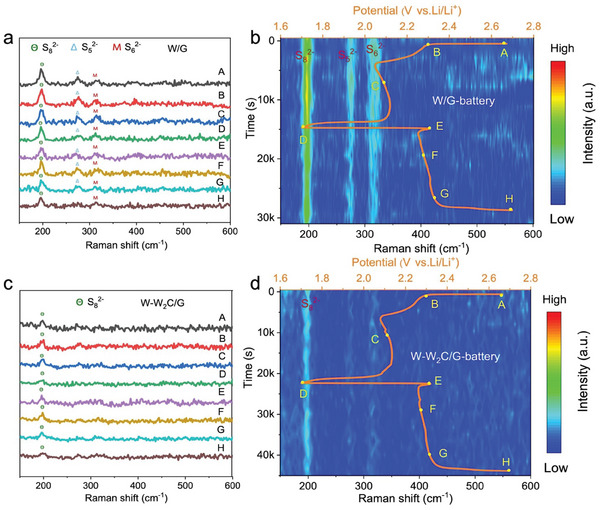
Suppressed LiPS shuttling demonstrated by in situ Raman measurements. In situ Raman spectroscopy at 0.2 C of the charge and discharge processes for Li–S batteries with a,b) W‐W_2_C/G and c,d) W/G interlayers.

In situ Raman characterization was conducted to monitor the electrochemical reactions in Li–S batteries, and in situ Raman spectra were collected on the separator at the lithium anode side upon different charge and discharge states (Figure S20, Supporting Information), where the intensity of Raman peaks indicates the concentration of LiPSs.^[^ ^33^, ^46^, ^47^ ^]^ As shown in **Figure** **5**, when W/G was used as the interlayer (Figure 5a,b), the observable signals at 197, 274, and 315 cm^−1^ were detected at the beginning of the discharge process, which were attributed to the S_8_^2−^, S_5_^2−^ and S_6_^2−^, respectively,^[^ ^47^, ^48^ ^]^ and these peaks persisted during the charging period, even they gradually decreased as the discharge progressing, indicating that the weak anchoring of LiPSs by W/G results in a significant amount of LiPSs penetrating across the separator to the lithium anode. In contrast, for the battery with W‐W_2_C/G interlayer, negligible LiPS signals were detected on the lithium anode (Figure 5c,d), which supports the restrained shuttling effect of LiPSs and the enhanced electrochemical performances above. In situ Raman characterization firmly verifies the synergistic effect between strong adsorption capability W_2_C and the high catalytic activity of metallic W to effectively suppress the shuttle effect of LiPSs and strengthen their reversible conversion during the charge/discharge processes. In addition, the surface of the lithium anode for the battery with W/G interlayer after 400 cycles at 3.0 C is highly rough and exhibits bulky deposition, resulting from the migration of numerous LiPSs, which is the main reason for the poor cycling performance with rapid capacity decay (Figure S21a, Supporting Information). In contrast, the battery with the W‐W_2_C/G interlayer shows a smooth and compact lithium anode surface with no significant dendritic growth (Figure S21b, Supporting Information), thereby improving the cycling stability. In order to provide a comprehensive understanding of the shuttle effect inhibition using the W‐W_2_C/G heterostructure, a LiPS‐diffusion experiment was conducted to assess the permeation of LiPSs through W‐W_2_C/G and W/G interlayers. The results depicted in Figure S22 (Supporting Information) demonstrate that the LiPSs were harder to permeate through the W‐W_2_C/G‐coated separator even after 12 h, indicating a notable suppression of LiPS shuttling in comparison to that for the W/G‐coated separator.

### 2.6 The General Applicability of Heterostructure Catalysts Prepared by Flash Joule Heating

**Figure 6:**
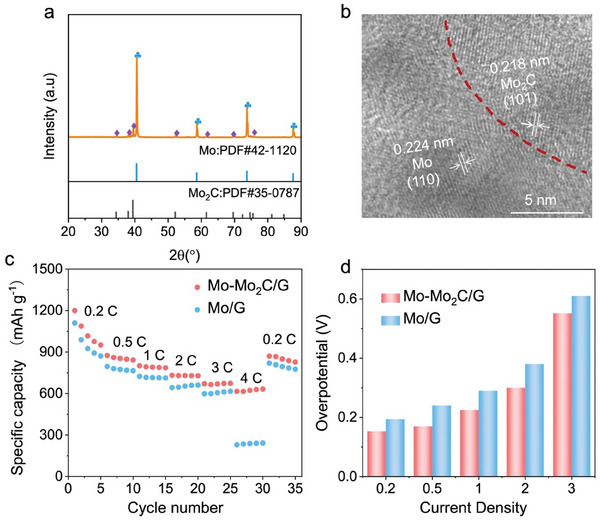
Materials characterization and electrochemical performance. a) XRD patterns of Mo‐Mo_2_C/G heterostructure. b) HRTEM image of the as‐prepared Mo‐Mo_2_C/G heterostructure. c) Rate performance. d) Overpotentials are calculated from the voltage difference between the charge and discharge plateaus in a cycle at rates of 0.2, 0.5, 1.0, 2.0, and 3.0 C.

To further probe into the general applicability of heterostructure catalysts in Li–S batteries, other heterostructure catalysts, such as Mo‐Mo_2_C/G, were synthesized using the flash Joule heating method. The XRD patterns, TEM, and HRTEM images presented in **Figures** **6**a,b and S23 (Supporting Information) provide strong evidence of the successful fabrication of the Mo‐Mo_2_C/G heterostructure, which is also used as the interlayer material. The battery with Mo‐Mo_2_C/G interlayer exhibited a positive rate performance as shown in Figure 6c; the specific capacities were approximately 1200, 874, 800, 732, 671, and 615 mAh g^−1^ at 0.2, 0.5, 1.0, 2.0, 3.0 and 4.0 C, superior to those of the battery with the Mo/G interlayer. Furthermore, the overpotentials of the battery with the Mo‐Mo_2_C/G interlayer are much lower than those of the control sample at all rates (Figure 6d and Figure S24, Supporting Information), indicating the excellent catalytic activity of the Mo‐Mo_2_C/G catalyst. These results give direct proof that introducing heterostructure catalysts prepared through the flash Joule heating method can greatly suppress the shuttle effect and elevate the electrochemical properties of Li–S batteries.

## 3 Conclusion

To summarize, this study introduces a universal approach for synthesizing heterostructure catalysts (e.g., W‐W_2_C/G and Mo‐Mo_2_C/G heterostructures) using the ultrafast flash‐Joule‐heating technique and examines their effectiveness as catalytic interlayers in Li–S batteries. Serial experimental measurements including in situ Raman performance and theoretical calculations verified that the W‐W_2_C heterostructure showed high adsorption capability towards LiPSs, and generated an internal electric field at the heterostructure interface to ensure the fast charge transfer, the greatly restrained shuttle effect of LiPSs, the enhanced kinetics of the SRR as well as the improved electrochemical performances. The W‐W_2_C interlayer enabled the Li–S battery to harvest a high capacity retention of 80.6% after 100 cycles at 0.2 C even at a high sulfur loading of 7.9 mg cm^−2^, and excellent stability for 1000 cycles at 3.0 C. This work provides a new strategy for the design of high‐performance electrocatalysts towards the practical Li–S batteries.

## Conflict of Interest

The authors declare no conflict of interest.

## Supporting information

Supporting Information

## Data Availability

The data that support the findings of this study are available from the corresponding author upon reasonable request.
