# Supplementary material for: Flash Joule Heating: A Promising Method for Preparing Heterostructure Catalysts to Inhibit Polysulfide Shuttling in Li–S Batteries
Source: Adv Sci (Weinh). 2024 Jul 16;11(35):2405351. doi: 10.1002/advs.202405351 (PMC11425280; doi:10.1002/advs.202405351)
Supplement: Supplementary file 1 — Supporting Information [file ADVS-11-2405351-s001.docx]

**Supporting Information**

Flash Joule Heating: A Promising Method for Preparing Heterostructure Catalysts to Inhibit Polysulfide Shuttling in Li-S Batteries

Huiyi Dong^1†^, Lu Wang^2†^, Yi Cheng^3^, Huiyue Sun^1^, Tianqi You^1^, Jingjing Qie^1^, Yifan Li^1^, Wuxing Hua^1^*, and Ke Chen^1^*

^1^ H. Dong, H. Sun, T. You, J. Qie, Y. Li, Prof. W. Hua, Prof. K. Chen

Center for the Physics of Low-Dimensional Materials, Henan Joint International Research Laboratory of New Energy Materials and Devices, School of Physics and Electronics, Henan University, Kaifeng, 475004, China

Email: wxhua@henu.edu.cn; kchen@henu.edu.cn

^2^ Dr. L. Wang

School of Materials Science and Engineering, Shandong University, Jinan 250061, P. R.

China.

^3^ Dr. Y. Cheng

Center for Nanochemistry, Beijing Science and Engineering Center for Nanocarbons, Beijing National Laboratory for Molecular Sciences, College of Chemistry and Molecular Engineering, Peking University, Beijing 100871, China.

^†^ These authors are equal main contributors


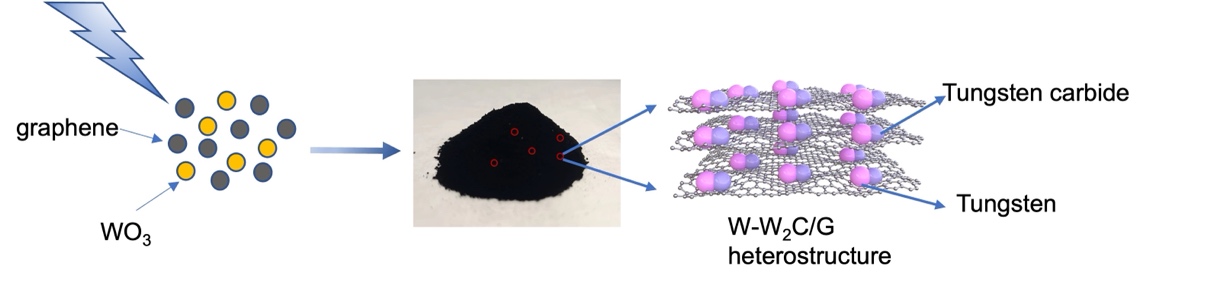


**Figure S1.** The fabrication process of W-W_2_C/G heterostructure catalysts using the flash-Joule-heating method.


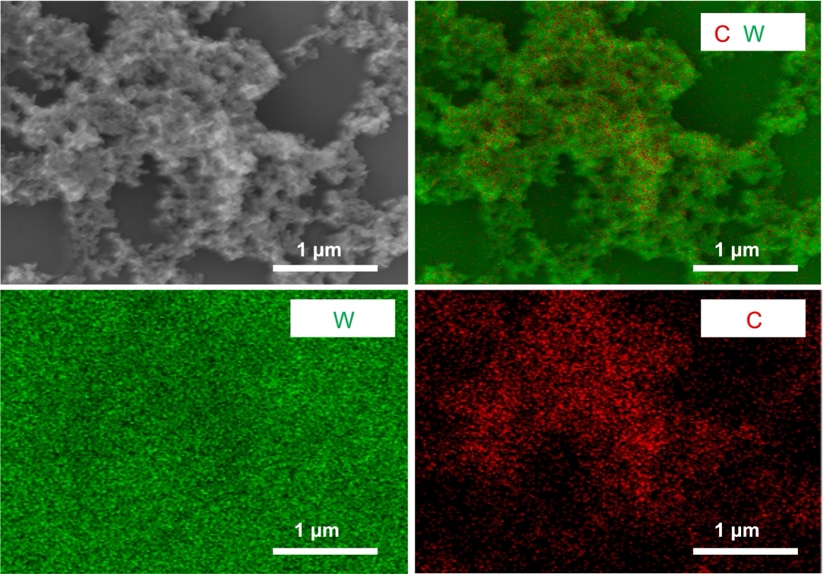


**Figure S2.** SEM image of W-W_2_C/G products and the corresponding elemental mapping images.


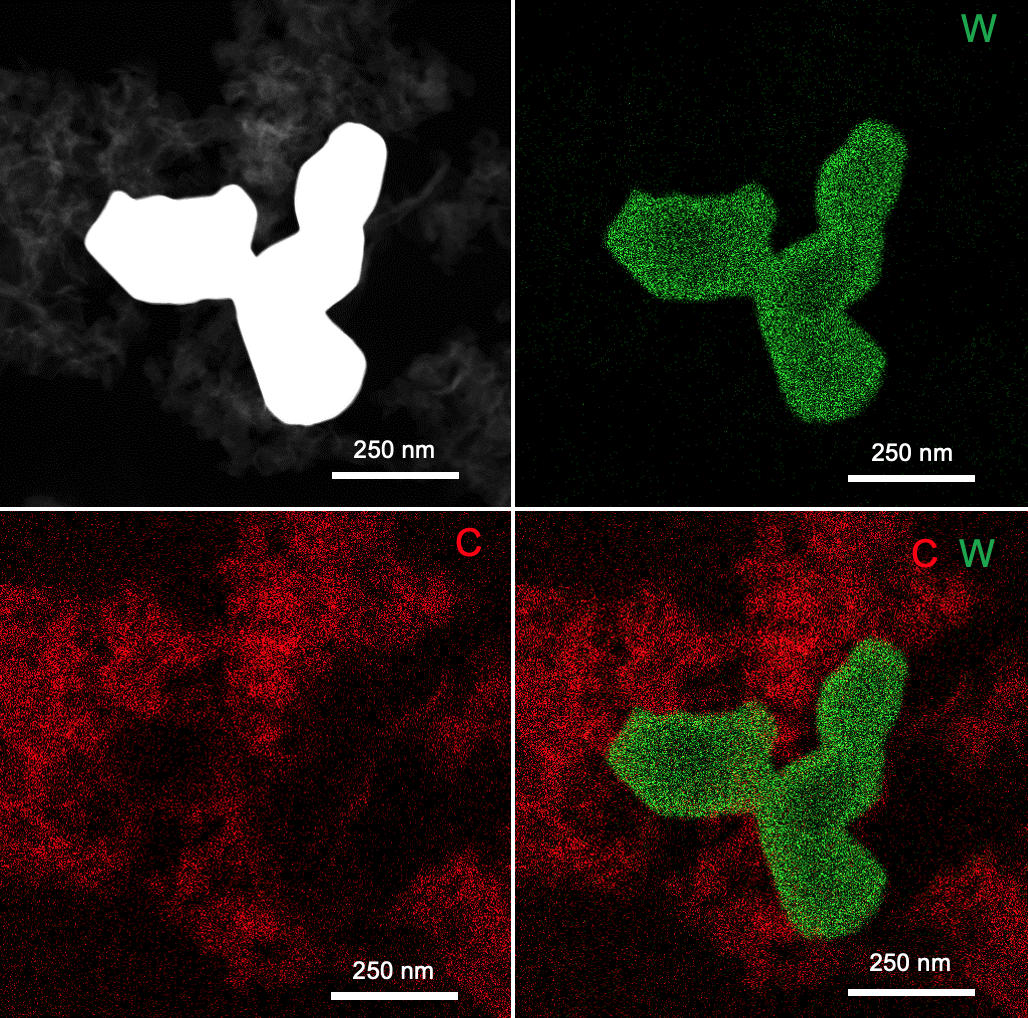


**Figure S3.** HAADF image of W-W_2_C/G products and the corresponding elemental mapping images.


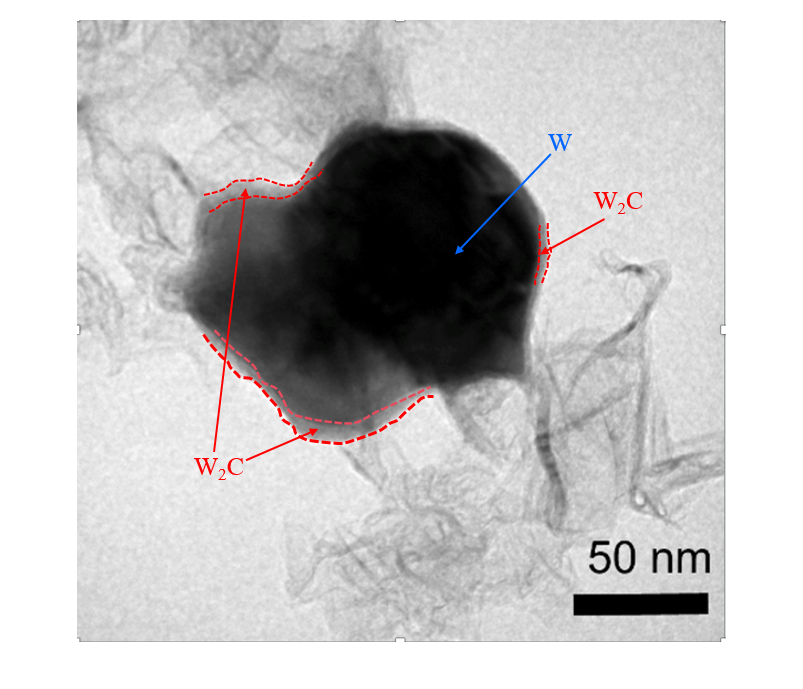


**Figure S4.** TEM image of the as-synthesized W-W_2_C heterostructure.


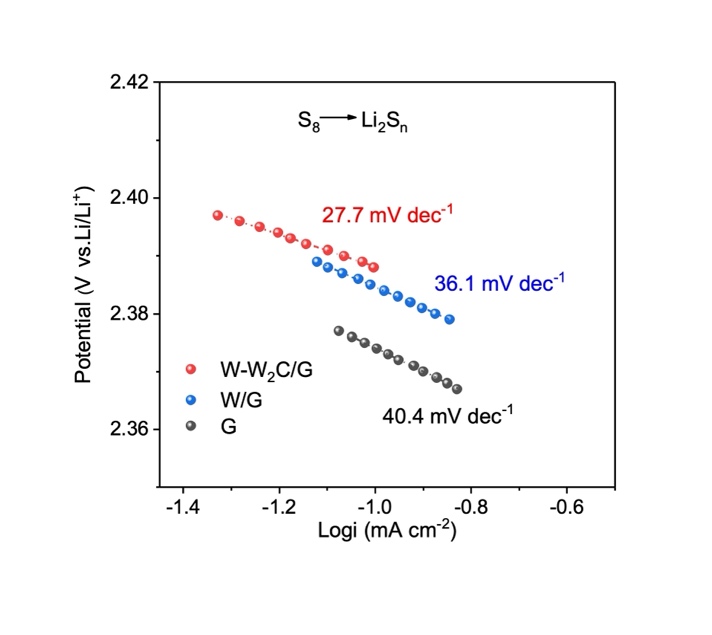


**Figure S5.** Tafel plots corresponding to the reduction of S_8_ to the Li_2_S_n_.


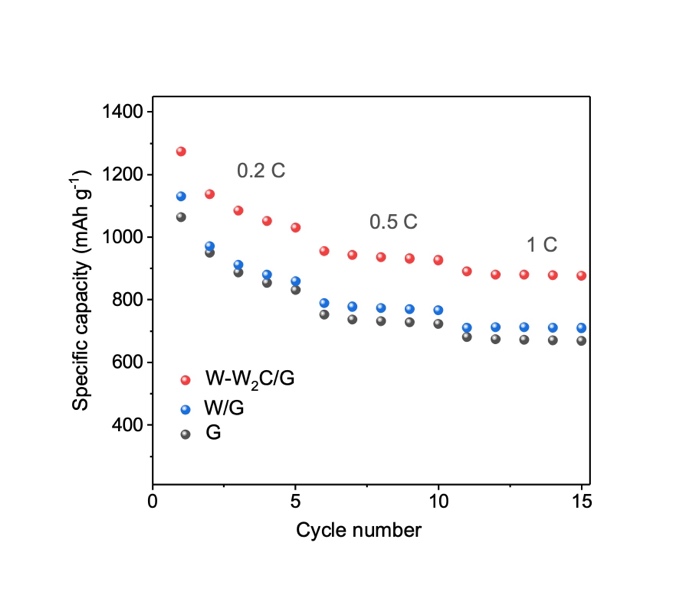


**Figure S6.** Rate performance for the batteries with W-W_2_C/G, W/G, and G interlayers.


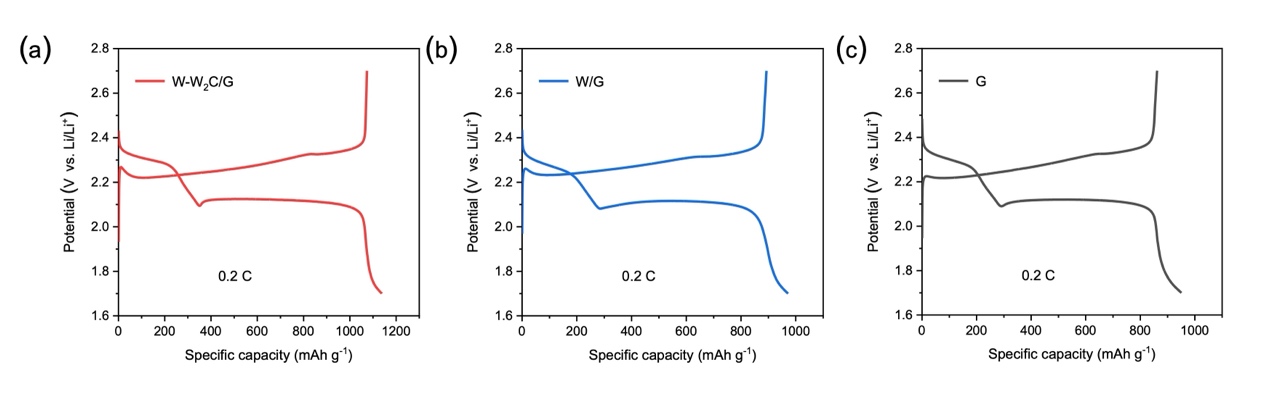


**Figure S7.** The discharge/charge curves of the batteries with (a) W-W_2_C/G and (b) W/G and (c) G interlayers at 0.2 C (data obtained from Figure S6). To obtain the charge efficiency, the discharge curves are taken from the second cycle, while the charge curves are from the third cycle of the three batteries at 0.2 C.

**
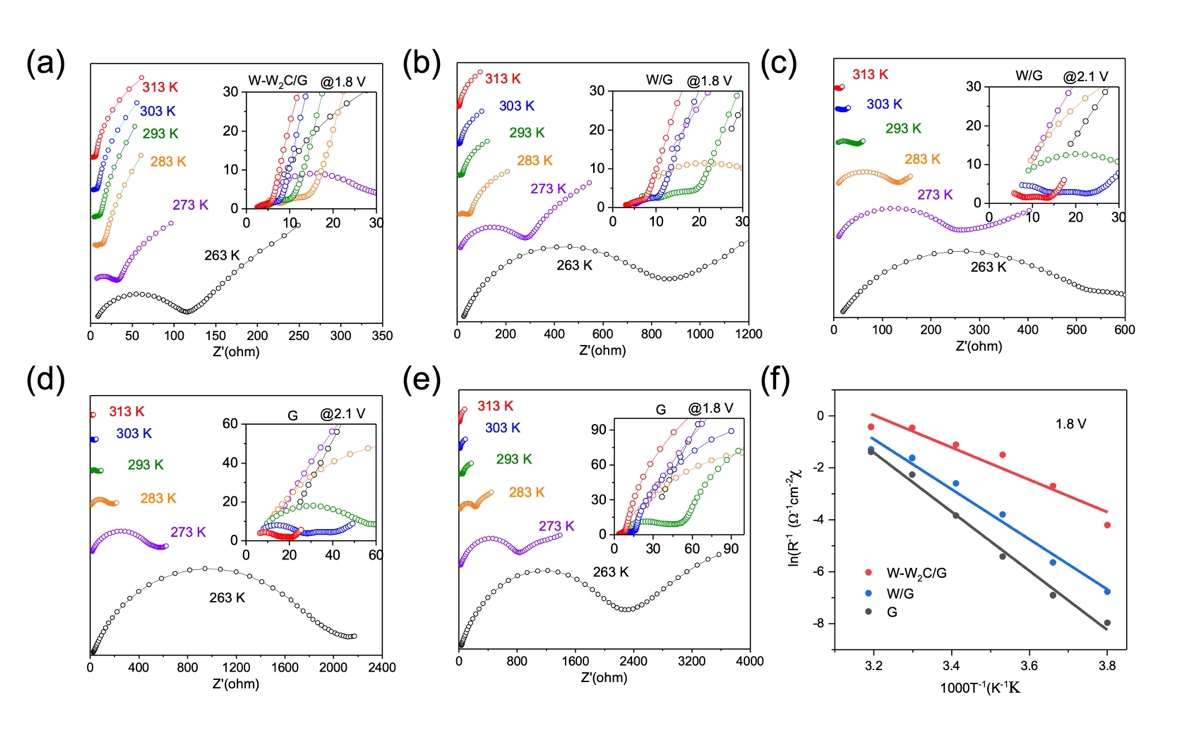
**

**Figure S8.** (a-e) EIS measurements at various temperatures at 2.1 V and 1.8 V. Inset: enlarged image of the EIS; (f) Arrhenius plots for the three batteries calculated from the EIS curves at 1.8 V.


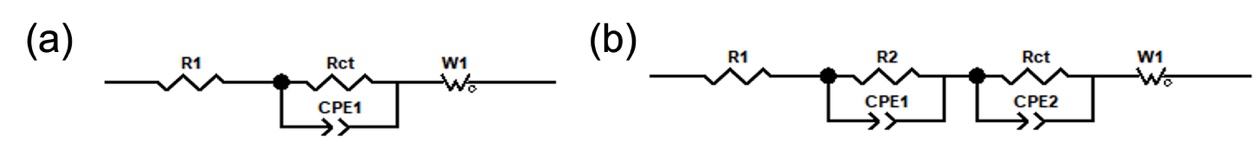


**Figure S9.** Simplified-contact Randles-equivalent circuits of EIS.


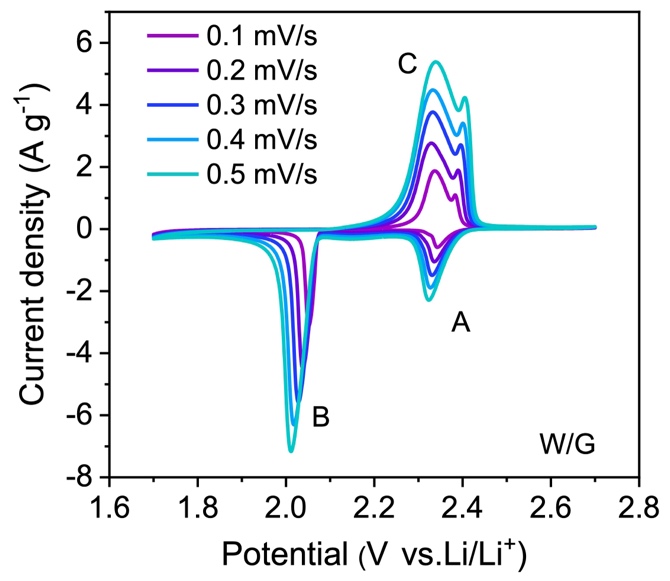


**Figure S10.** CV curves of the battery with W/G interlayer at various scan rates


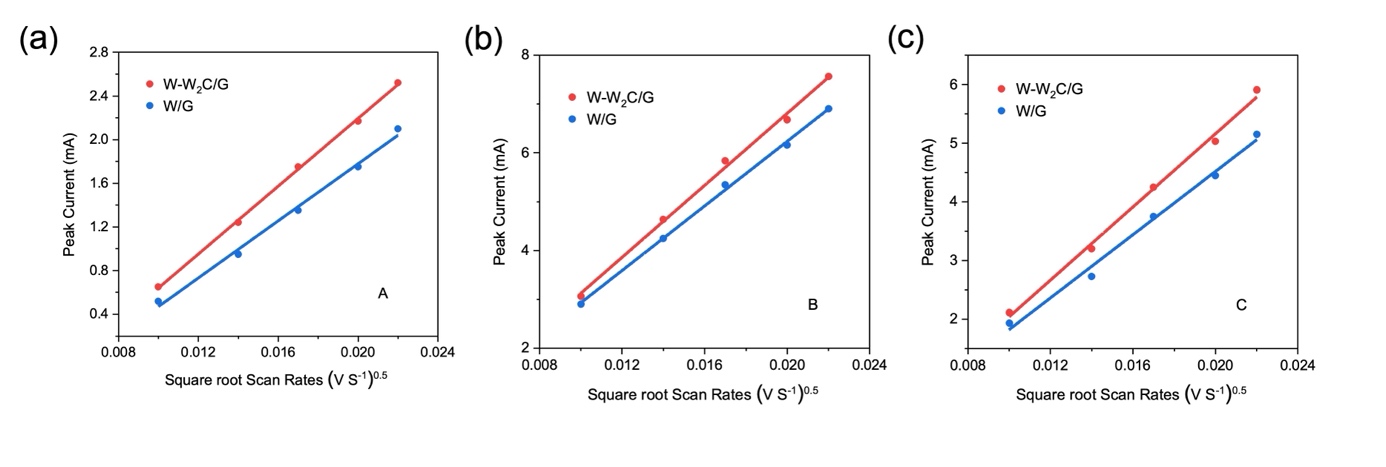


**Figure S11.** Linear fitting of the peak currents versus the square roots of the scan rate: (a) cathodic peak A; (b) cathodic peak B; (c) anodic peak C.


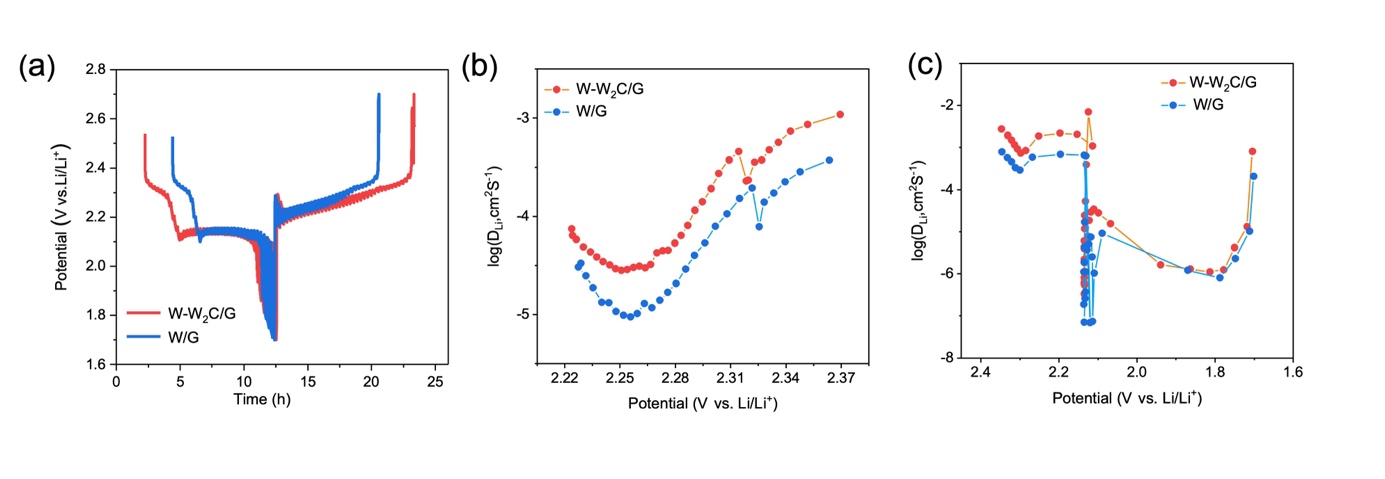


**Figure S12.** (a) Temporal voltage evolution in GITT measurement and the corresponding Li-ion diffusion coefficients in the charging process (b), and the discharging process (c).


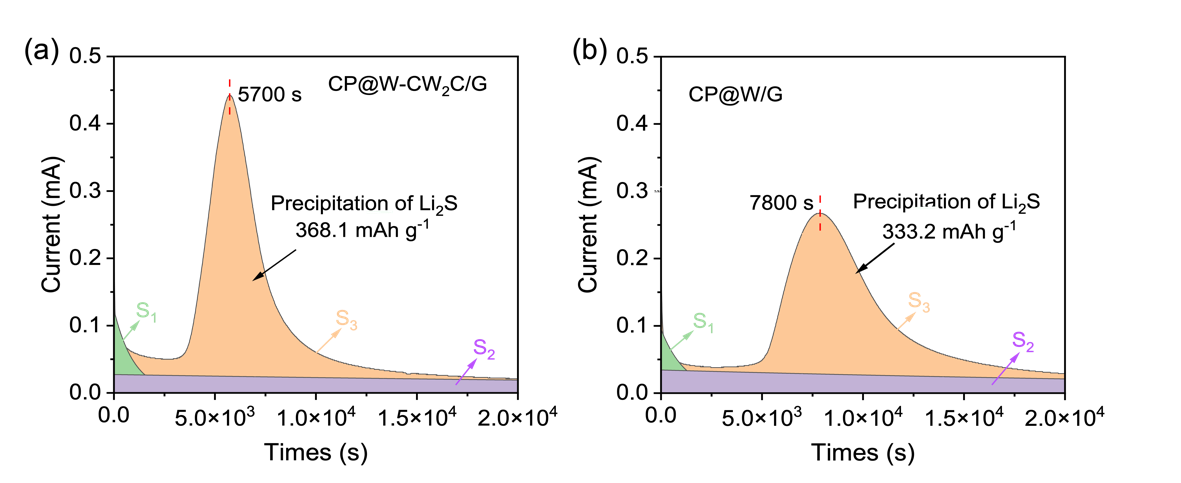


**Figure S13.** Potentiostatic discharge profiles for (a) CP@W-W_2_C/G cell and (b) CP@W/G cell at 2.05 V.

The area under the potentiostatic discharge curves could be mathematically modeled as three parts (S_1_, S_2_, S_3_): the areas of S_1_ and S_2_ respectively represent the reduction of Li_2_S_8_ and Li_2_S_6_, and the area of S_3_ represents the precipitation of Li_2_S. The conversion capacity is obtained based on the Faraday’s law: the whole integral area of the potentiostatic discharge curve is denoted as S_0_, and the weight of sulfur in the Li_2_S_8_ electrolyte is denoted as W. Thus, the capacity of Li_2_S precipitation can be calculated by the formula (S_0_-S_1_-S_2_)/3600/W.

**
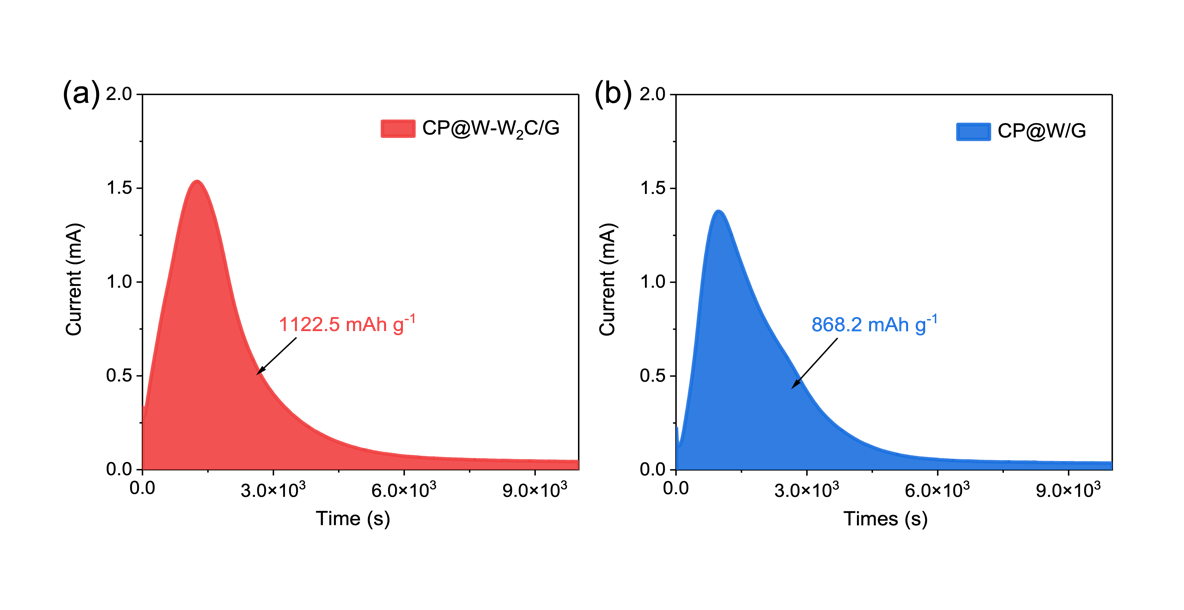
**

**Figure S14.** Potentiostatic charge profiles for the (a) CP@W-W_2_C/G cell and (b) CP@W/G cell at 2.45 V.

**
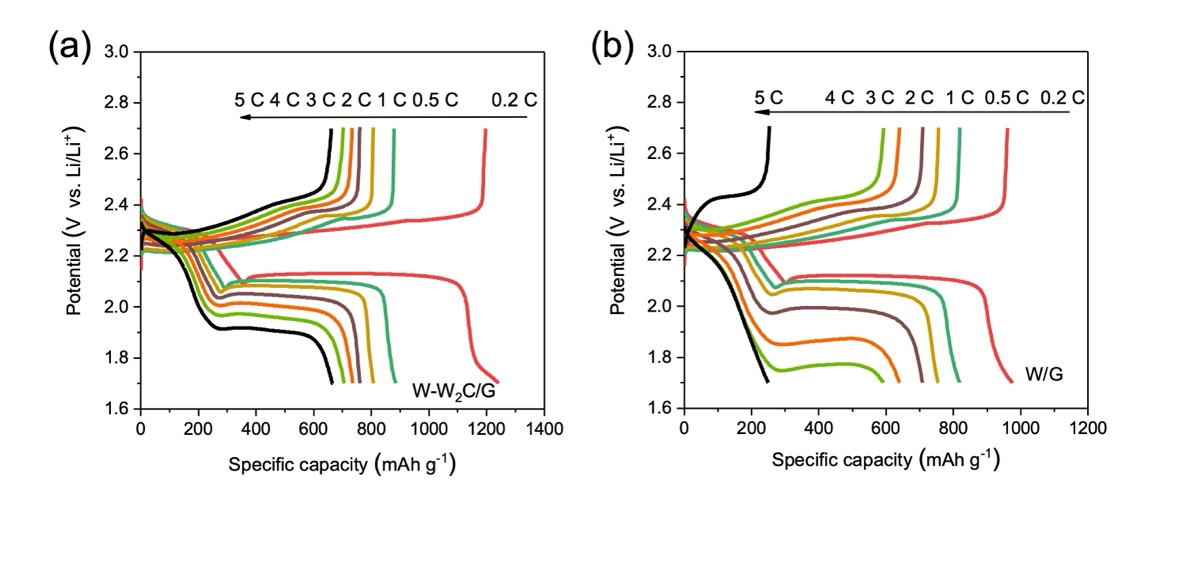
**

**Figure S15.** Galvanostatic charge-discharge profiles of the batteries with (a) W-W_2_C/G and (b) W/G interlayers at different rates.


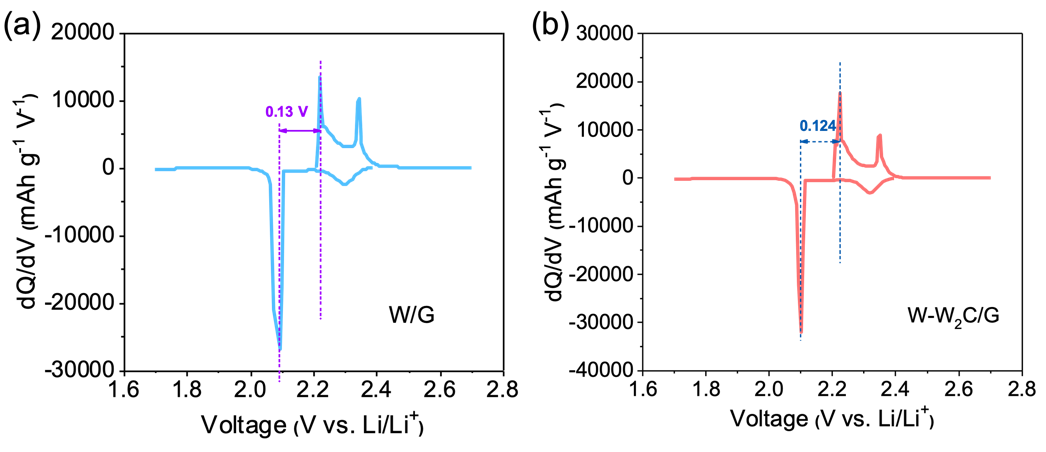


**Figure S16.** The differential plots corresponding to the charge/discharge profiles of batteries with W/G and W-W_2_C/G interlayers.

**
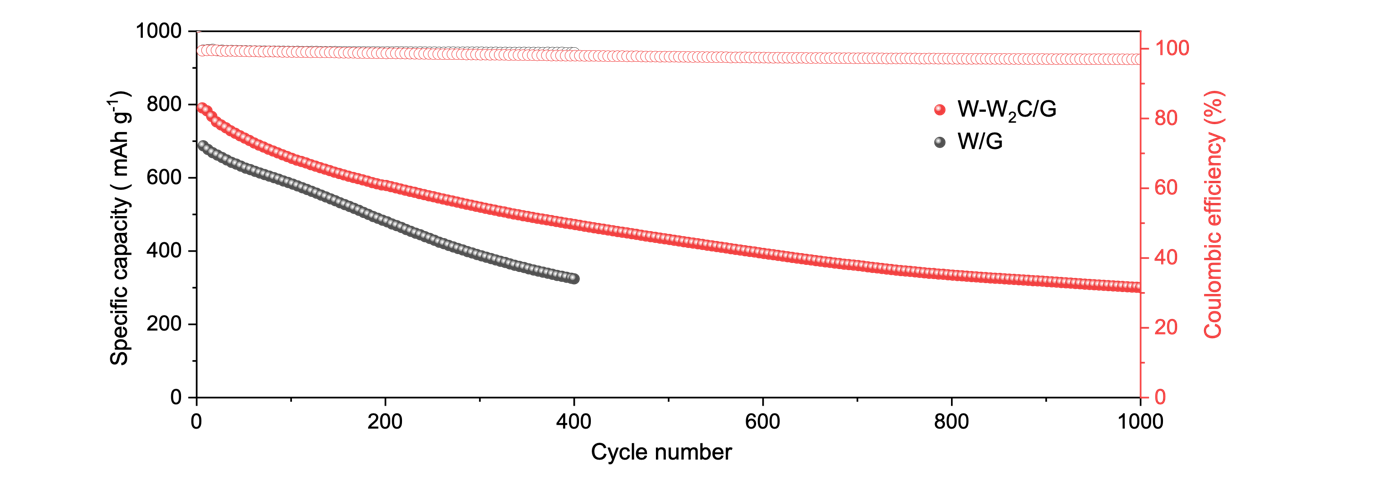
**

**Figure S17.** Long-term cycling stability at 3.0 C for the battery with W-W_2_C/G and W/G interlayers (sulfur loading: 1.0 mg cm^−2^)


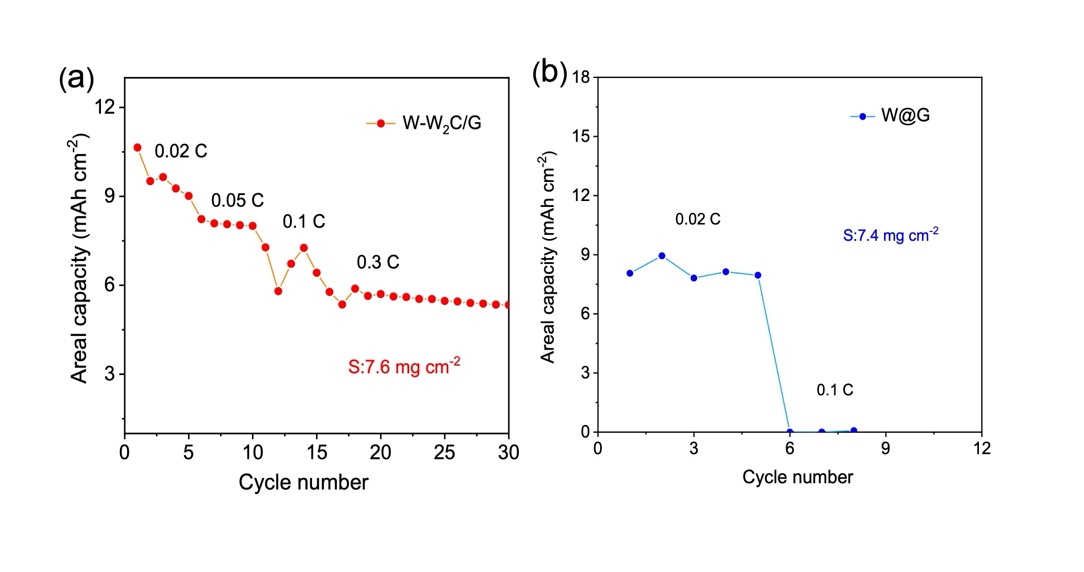


**Figure S18.** (a) Rate performance of Li–S batteries with (a) W-W_2_C/G interlayer (sulfur loading: 7.6 mg cm^−2^) and (b) W/G interlayer (sulfur loading: 7.4 mg cm^−2^).


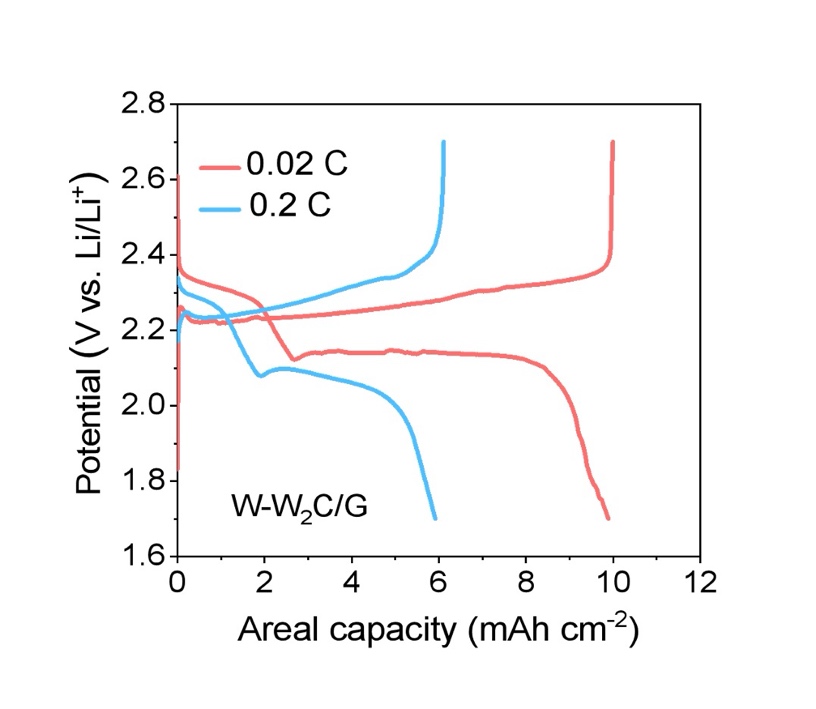


**Figure S19.** The charge-discharge curves for the battery with W-W_2_C/G interlayer with a high sulfur mass loading of 7.9 mg cm^-2^ at 0.02 and 0.2 C.


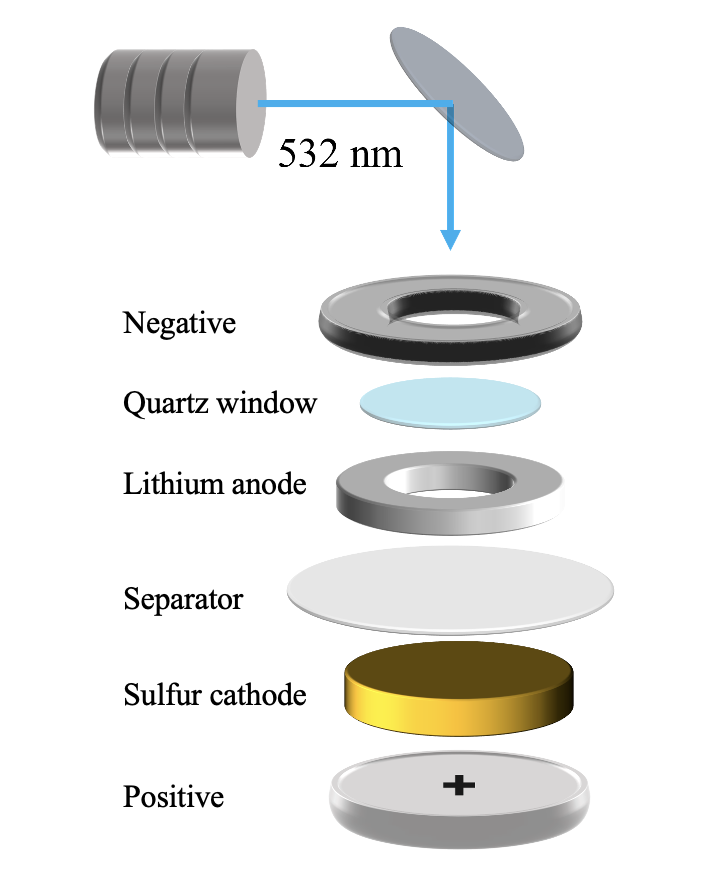


**Figure S20.** Cell configuration with a quartz window for *in-situ* Raman analysis.


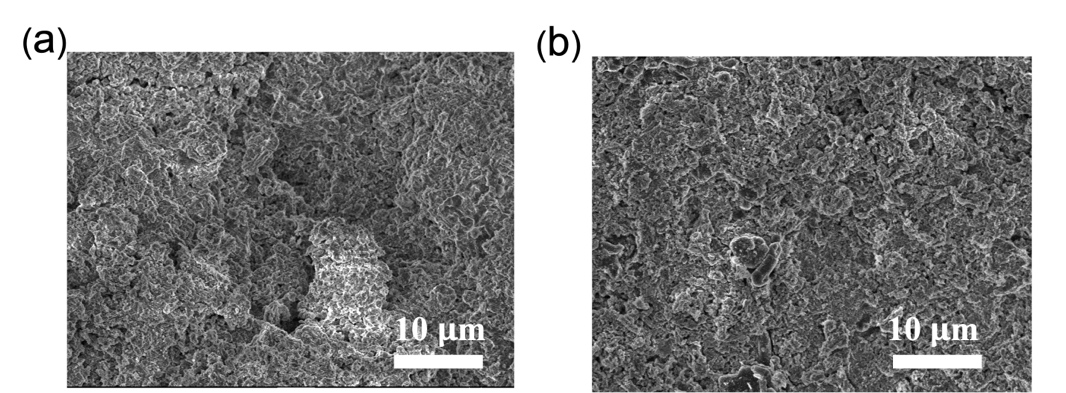


**Figure S21.** SEM images of cycled lithium anodes for the batteries with (a) W/G and (b) W-W_2_C/G interlayers after 400 cycles.


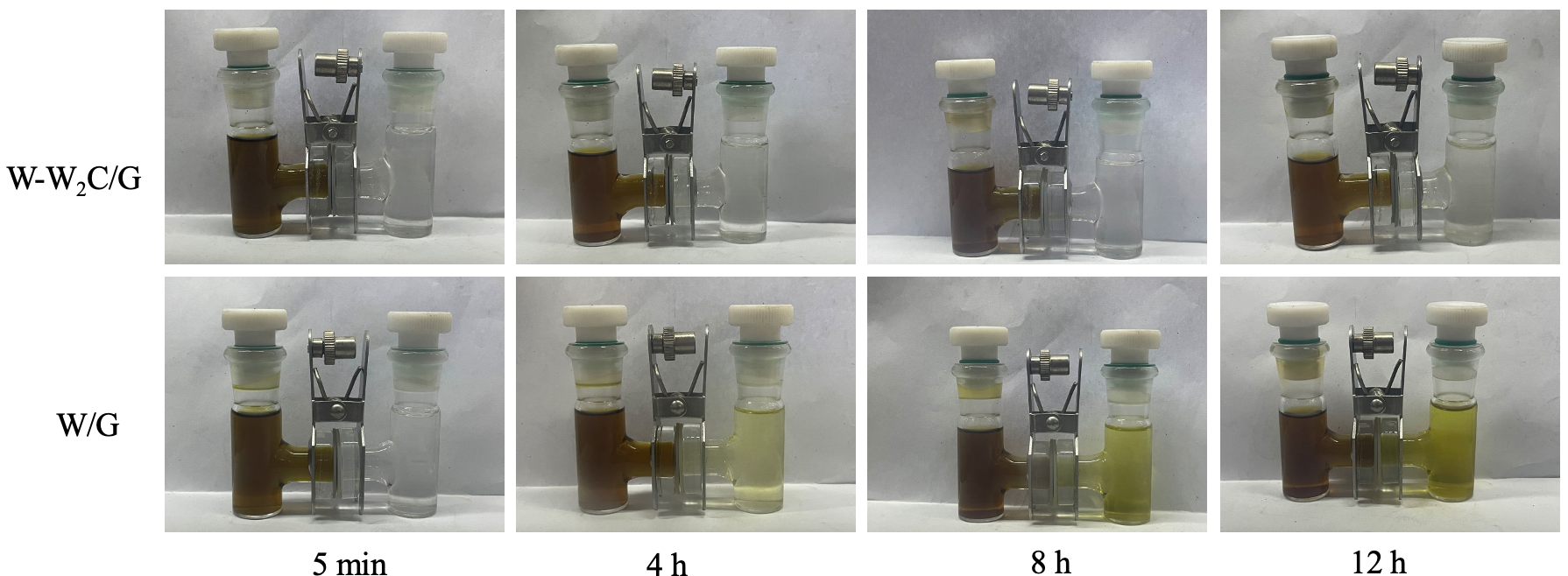


**Figure S22.** Polysulfide permeation measurements with H-type electrolytic cells with W-W_2_C/G-coated separator, and W/G-coated separator.


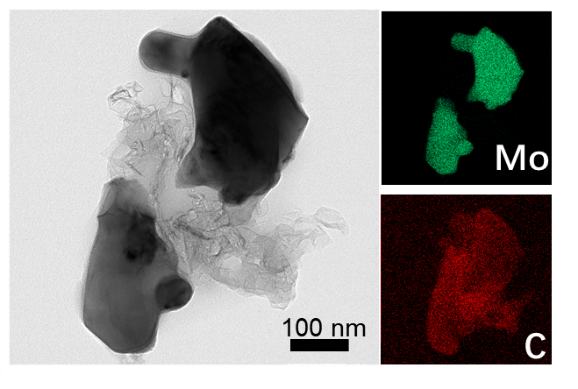


**Figure S23.** TEM images of Mo-Mo_2_C/G and corresponding elemental mapping images of Mo-Mo_2_C/G.


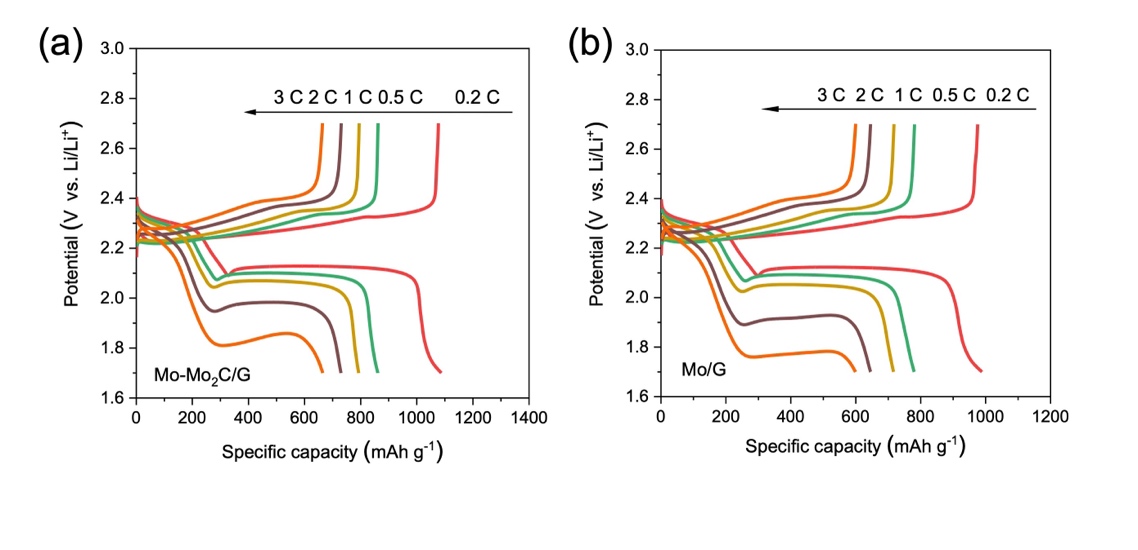


**Figure S24.** Galvanostatic charge-discharge profiles of the batteries with (a) Mo-Mo_2_C/G and (b) Mo/G interlayers at different rates.


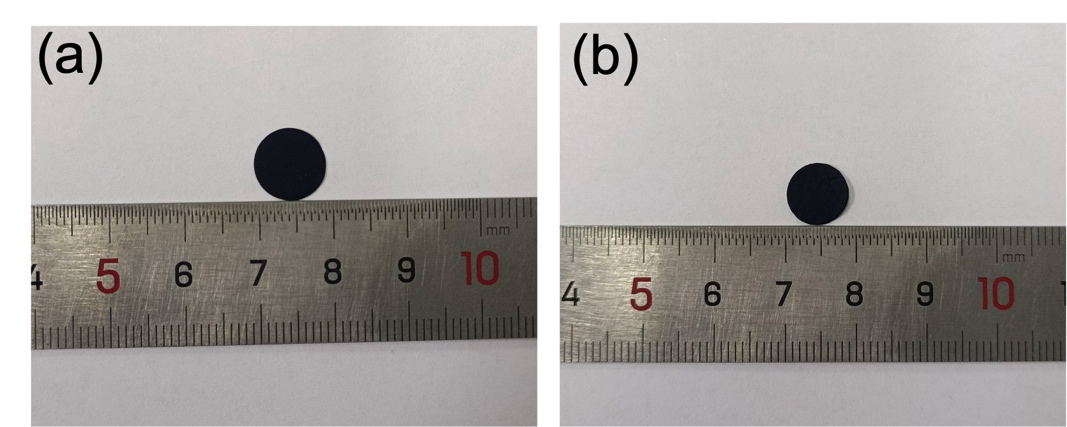


**Figure S25.** The digital photographs of freestanding high sulfur-loaded cathodes (a) before and (b) after vacuum drying.

**Table S1.** Comparison of electrochemical properties of recent publications in Li-S batteries with high sulfur loadings and low electrolyte/sulfur ratios.

| Cathode  materials | Sulfur  loading (mg cm^−2^) | E/S ratio  (μL mg^−1^) | Capacity  (mAh cm^−2^) | Reference |
| --- | --- | --- | --- | --- |
| W-W_2_C/G | 7.9 | 9 | 10.9 (Initial @0.02C)  5.0 (100 cycles @0.2C) | **This work** |
| VC-1/PP^[1]^ | 5.3 | 7 | 3.7 (Initial @0.2C)  2.78 (100 cycles @0.2C) | [1] |
| CoFe-MCS^[2]^ | 5.3 | 9.4 | 5.2 (Initial @0.1C)  4.0 (45 cycles @0.1C) | [2] |
| S@Ni-MOF-1D^[3]^ | 6.7 | 7.6 | 6.63 (Initial @0.1C) | [3] |
| Co-TiN/C^[4]^ | 4 | 8.5 | 4.7 (Initial @0.05C)  2.49 (100 cycles @0.3C) | [4] |
| SA-Fe/Fe_2_N@NG^[5]^ | 5 | / | 6.0 (Initial @0.1C)  5.02 (100 cycles @0.1C) | [5] |
| G@ppy-por^[6]^ | 5 | 6.8 | 4.7 (Initial @0.2C)  4.0 (50 cycles @0.2C) | [6] |
| PZI@PP^[7]^ | 5.8 | 7 | 6.45 (Initial @0.1C)  5.3 (300 cycles @0.1C) | [7] |
| Co/MoN@PP^[8]^ | 4.92 | 10 | 7.72 (Initial @0.05C)  4.62(60 cycles @0.1C) | [8] |
| FeCoPS_3_/NC^[9]^ | 6.5 | / | 4.76 (Initial @0.1C)  4.33 (100 cycles @0.1C) | [9] |
| CuL^[10]^ | 10.4 | 6 | 9.62 (Initial @0.05C)  7.8 (70 cycles @0.05C) | [10] |

**Experimental Section:**

***Synthesis of*** ***W-W_2_C/G heterostructure catalysts***: Tungsten oxide (Sigma-Aldrich, 99.9%) and graphene (Carmery, 99.9%) were mixed with a mass ratio of 1:1, which was fully grounded in a mortar. Then the powder was put into a Joule thermal instrument for rapid and massive synthesis of the W-W_2_C/G heterostructure catalysts. W/G composites were prepared by mixing tungsten powder (Macklin, 99.9%) and graphene (Carmery, 99.9%) with a mass ratio of 1:2, which was fully grounded in a mortar.

***Synthesis of CNTs/S cathodes***: The carbon nanotubes/sulfur (CNTs/S) composites were prepared by a simple melt-diffusion method according to the previous report. Typically, the CNTs (Aladdin, 95%) and sulfur (Kermel, 99.5 %) were mixed with a ratio of 3:7, which was grounded uniformly, and heated in an oven at 155 °C for 12 h. The sulfur content in the CNTs/S composites was approximately ~70 wt%. For the CNTs/S cathodes, 80 wt% CNTs/S composites, 10 wt% conductive agents (CNTs), and 10 wt% polyvinylidene difluorides (PVDF) were mixed in N-methyl-2-pyrrolidone (NMP), which was stirred for 3 hours to form a slurry. Then, the slurry was coated on a carbon-coated aluminum foil current collector and vacuum-dried at 55 °C for 8 h.

***Synthesis of CNTs/S cathodes with W-W_2_C/G and W/G interlayers***: The W-W_2_C/G or W/G composites were dispersed in NMP to form a slurry, which was then coated on the prepared CNTs/S cathodes. After drying, the CNTs/S cathode with W-W_2_C/G or W/G interlayer was cut into circles of a diameter of 10 mm (denoted CNTs/S@W-W_2_C/G or CNTs/S@W/G). The areal sulfur loading was about 1.0 mg cm^−2^, and the interlayer areal loading was approximately 0.13 mg cm^−2^.

***Preparation of*** ***freestanding high sulfur-loaded cathodes***: 80 wt% CNT/S composite, 15wt% W-W_2_C/G or W/G as a conductive agent, and 5 wt% polytetrafluoroethylene (PTFE) as binder were dissolved in ethanol, which was evaporated through hair dryer until it turned into silly putty-like slurry. The obtained slurry was rolled into a thin sheet and cut into circular electrodes with a diameter of 10 mm, which shrank to ~ 8.5 mm (**Figure S25**) after vacuum drying at 55 °C for 8 h.

***Battery assembly and electrochemical measurements***: Standard CR2032 coin battery was assembled in an Ar-filled glovebox with CNTs/S@W-W_2_C/G or CNTs/S@W/G as the cathode, lithium foil as the anode, Celgard 2500 as the separator, and 1.0 M LiTFSI in DOL: DME =1:1 vol% with 1.0 % LiNO_3_ as the electrolyte. The electrolyte/sulfur (E/S) ratio was 30:1 for the normal Li-S batteries and 9:1 for the batteries with high sulfur loadings. The thickness of the lithium metal anode was 200 μm, and the ratios of N/P were 27.6 and 3.5 with sulfur loading of 1.0 and 7.9 mg cm^−2^, respectively. The galvanostatic discharge/charge measurements were conducted using a Neware battery test system and the voltage range was 1.7-2.7 V (*vs* Li^+^/Li). CV curves were obtained using a DongHua workstation at a scan rate of 0.1 mV s^-1^, and EIS measurements at various temperatures were conducted with a frequency range of 0.1-10^4^ Hz on the same workstation. GITT measurements were performed by discharging/charging the battery with a 10 min current pulse at 0.1 C, followed by a 5 min rest to collect the potential response. The diffusion coefficients of Li ions can be determined using Equation (1)^[11]^.

$$D_{\mathrm{Li}}=\frac{4}{\pi\tau}{(\frac{mV_{m}}{\mathrm{SM}})}^{2}{[\frac{\Delta E_{s}}{\Delta E_{t}}]}^{2}, t\ll L^{2}/D_{Lⅈ}$$

(1)

Where D_Li_ is the Li^+^ diffusion coefficient, L is the Li^+^ diffusion distance, τ is the relaxation time, m is the mass of the electrode material, M is the corresponding molar mass, ΔE_s_ is the total voltage change caused by the pulse, and ΔE_t_ is the voltage change during constant current charge or discharge.

***Assembly of the*** ***Li_2_S_6_–Li_2_S_6_ Symmetric Batteries***: The Li_2_S_6_ electrolyte (0.2 M) was purchased from DODOCHEM. W-W_2_C/G or W/G composites were ultrasonically dispersed in NMP, and then the dispersion was dropped onto the carbon paper, which was cut into circular pellets with a diameter of 10 mm after drying for use as the electrodes. Two same electrodes were assembled into a standard CR2032 cell with a polypropylene (PP) membrane as the separator and 40 µl Li_2_S_6_ electrolyte (20 µl for each side of the separator).

***Measurement for the deposition of Li_2_S***: The battery was assembled by using W-W_2_C/G or W/G composites loaded onto carbon paper with a diameter of 10 mm as the cathode, lithium foil as the anode, 20 µl Li_2_S_8_ electrolyte (0.2 M) purchased from DODOCHEM as catholyte, and 20 µl 1.0 M LiTFSI in DOL: DME =1:1 vol% with 1.0 % LiNO_3_ as the anolyte. The assembled batteries were discharged galvanostatically at a current of 0.112 mA until the voltage dropped to 2.06 V, then discharged potentiostatically at 2.05 V for Li_2_S deposition until the current fell below 10^-5^ A.

***In Situ Raman Spectroscopy***: Li-S battery with a quartz window used for in situ Raman spectroscopy analysis at 532 nm Laser was respectively assembled by coupling CNTs/S@W-W_2_C/G or CNTs/S@W/G as the cathode, 1.0 M LiTFSI in DOL: DME =1:1 vol% with 1.0 % LiNO_3_ as the electrolyte, and lithium metal as the anode. The battery was tested at a current of 0.2 C.

***The shuttle constant ks:*** The shuttle constants (*k*s, h-1) of the three batteries were determined by the charge-discharge curves at 0.2 C (**Figure S5**) using the following formulae^[12]^:

$\frac{k_{s}q_{H}[S_{\mathrm{total}}]}{I_{C}}=f_{C}$ (2)

$H_{OC}=\frac{Q_{H}^{applied}-Q_{H}^{acc}}{Q_{H}^{acc}}=-\frac{1}{f_{C}}\ln\left( 1-f_{C} \right)-1$ (3)

Where *f*C is the charge-shuttle factor; [Stotal] is the total sulfur mass in the battery, which is ~ 0.8 mg for all samples in this work; *q*H is the high plateau capacity (419 mAh g-1); *I*C is the current density at 0.2 C; $Q_{H}^{applied}$ is the applied high plateau charge capacity and is simplified here to the maximum charge capacity; $Q_{H}^{acc}$ is the high plateau accumulated charge capacity that is simplified here to the practical charge capacity.

***Structure Characterization***: The morphologies and phase structures of the samples in this work were probed by SEM (JSM-7610F Plus, JEOL) equipped with an EDS system, XRD (D8-ADVANCE, Bruker at room temperature), Raman spectroscopy (Reflex inVia with a 532 nm laser as the excitation source), and TEM (JEM-F200, JEOL, operating at 200 KV).

***Computational methods***: The density functional theory (DFT) calculations were applied by using the Vienna Ab initio Simulation Package (VASP) with Perdew-Burke-Ernzerhof (PBE). W (110) and W_2_C (102) surfaces were selected for calculation. A Monkhorst-Pack 5 × 5 × 1 grid in k-space were used. The adsorption energy (Eads) of Li_2_S_4_ on W (110) and W_2_C (102) surfaces was calculated with a plane-wave cutoff energy of 550 eV and a vacuum height of 10 Å^[13,14]^. The electronic energy was considered self-consistent when the energy change was smaller than 10^-5^ eV. A geometry optimization was considered convergent when the energy change was smaller than 10^-4^ eV Å^−1^. The climbing-image nudged elastic band (CI-NEB) method was performed to determine the energy barrier for the decomposition of Li and Li_2_S_4_ on W_2_C (102). The decomposition energy barrier of Li_2_S_4_ in W (110) and W_2_C (102) surfaces is calculated in the same way^[15,16]^.

**Reference:**

[1] L. Chen, Y. Sun, X. Wei, L. Song, G. Tao, X. Cao, D. Wang, G. Zhou, Y. Song, *Advanced Materials* **2023**, 35, 2300771.

[2] Z. Shi, Z. Sun, J. Cai, Z. Fan, J. Jin, M. Wang, J. Sun, *Advanced Functional Materials* **2021**, 31, 2006798.

[3] D. Yang, Z. Liang, P. Tang, C. Zhang, M. Tang, Q. Li, J. J. Biendicho, J. Li, M. Heggen, R. E. Dunin‐Borkowski, M. Xu, J. Llorca, J. Arbiol, J. R. Morante, S. Chou, A. Cabot, *Advanced Materials* **2022**, 34, 2108835.

[4] Q. Liu, Y. Wu, D. Li, Y. Peng, X. Liu, B. Li, J. Huang, H. Peng, *Advanced Materials* **2023**, 35, 2209233.

[5] C. Ma, Y. Zhang, Y. Feng, N. Wang, L. Zhou, C. Liang, L. Chen, Y. Lai, X. Ji, C. Yan, W. Wei, *Advanced Materials* **2021**, 33, 2100171.

[6] C.-X. Zhao, X.-Y. Li, M. Zhao, Z.-X. Chen, Y.-W. Song, W.-J. Chen, J.-N. Liu, B. Wang, X.-Q. Zhang, C.-M. Chen, B.-Q. Li, J.-Q. Huang, Q. Zhang, *Journal of the American Chemical Society*. **2021**, 143, 19865.

[7] G. Li, F. Lu, X. Dou, X. Wang, D. Luo, H. Sun, A. Yu, Z. Chen, *Journal of the American Chemical Society*. **2020**, 142, 15200.

[8] Y. Kong, L. Wang, M. Mamoor, B. Wang, G. Qu, Z. Jing, Y. Pang, F. Wang, X. Yang, D. Wang, L. Xu, *Advanced Materials* **2023**, 2310143.

[9] H. Li, M. Chuai, X. Xiao, Y. Jia, B. Chen, C. Li, Z. Piao, Z. Lao, M. Zhang, R. Gao, B. Zhang, Z. Han, J. Yang, G. Zhou, *Journal of the American Chemical Society*. **2023**, 145, 22516.

[10] Z. Yu, X. Huang, M. Zheng, S. Zhang, Y. Yang, J. Lu, *Advanced Materials* **2023**, 35, 2300861.

[11] Z. Zhou, Z. Chen, H. Lv, Y. Zhao, H. Wei, B. Chen, Y. Wang, *Energy Storage Materials* **2022**, 51, 486.

[12] W. Hua, T. Shang, H. Li, Y. Sun, Y. Guo, J. Xia, C. Geng, Z. Hu, L. Peng, Z. Han, C. Zhang, W. Lv, Y. Wan, *Nature Catalysis* **2023**, 6, 174.

[13] J. Pu, W. Gong, Z. Shen, L. Wang, Y. Yao, G. Hong, *Advanced Science* **2022**, 9, 2104375.

[14] T. Xiao, L. Zhao, H. Ge, M. Yang, W. Liu, G. Li, M. Ren, X. Zhang, Z. Zhou, *Chemical Engineering Journal* **2022**, 439, 135790.

[15] X.-X. Yang, X.-T. Li, C.-F. Zhao, Z.-H. Fu, Q.-S. Zhang, C. Hu, *ACS Applied Materials & Interfaces* **2020**, 12, 32752.

[16] T. Zhou, W. Lv, J. Li, G. Zhou, Y. Zhao, S. Fan, B. Liu, B. Li, F. Kang, Q.-H. Yang, *Energy and Environmental Science*. **2017**, 10, 1694.
